# A Rule-Based Spatial Reasoning Approach for OpenStreetMap Data Quality Enrichment; Case Study of Routing and Navigation

**DOI:** 10.3390/s17112498

**Published:** 2017-10-31

**Authors:** Amin Mobasheri

**Affiliations:** GIScience research group, Institute of Geography, Heidelberg University, Im Neuenheimer Feld 348, 69120 Heidelberg, Germany; a.mobasheri@uni-heidelberg.de; Tel.: +49-6221-54-5547

**Keywords:** data mining, OpenStreetMap, data quality enrichment, routing, crowdsourced geographic information, VGI

## Abstract

Finding relevant geospatial information is increasingly critical because of the growing volume of geospatial data available within the emerging “Big Data” era. Users are expecting that the availability of massive datasets will create more opportunities to uncover hidden information and answer more complex queries. This is especially the case with routing and navigation services where the ability to retrieve points of interest and landmarks make the routing service personalized, precise, and relevant. In this paper, we propose a new geospatial information approach that enables the retrieval of implicit information, i.e., geospatial entities that do not exist explicitly in the available source. We present an information broker that uses a rule-based spatial reasoning algorithm to detect topological relations. The information broker is embedded into a framework where annotations and mappings between OpenStreetMap data attributes and external resources, such as taxonomies, support the enrichment of queries to improve the ability of the system to retrieve information. Our method is tested with two case studies that leads to enriching the completeness of OpenStreetMap data with footway crossing points-of-interests as well as building entrances for routing and navigation purposes. It is concluded that the proposed approach can uncover implicit entities and contribute to extract required information from the existing datasets.

## 1. Introduction

Sound decision-making in the geographical domain involves answering to complex queries, which requires inferring facts from available geospatial data sources. Meanwhile, the amount of available data has been rapidly growing, due, among other phenomena, to the increasing dissemination of digital sensors, smart phones, crowdsourcing applications, and social media, etc. The phenomenon of crowdsourcing in general and Volunteered Geographic Information (VGI), in particular, is a new paradigm that could help to enrich the already existing frameworks in GIScience (e.g., routing services). A well-known example is OpenStreetMap (OSM), which has now become an experimental platform to study the VGI phenomena and demonstrate all of the opportunities of VGI (as a subset of open geospatial data) for a plethora of applications, especially in urban studies [1,2]. With these new promises, users are expecting that not only they will have access to large datasets, but more importantly, they will be able to pose more complex queries and infer more information than ever. However, the quality of VGI data is questionable [3,4,5] and methods need to be investigated and developed for data enrichment [6]. Data completeness is one of the spatial data quality elements according to ISO 19157 standard [7], which refers to the presence or lack of certain information in a dataset. Based on the results of OSM data quality assessment in terms of data completeness, we found out that there are missing objects (e.g., footway crossings), which are required for proper and efficient pedestrian/wheelchair routing and navigation [8,9,10]. Footway crossings are defined as perpendicular sections of footway at a crossing point between two sidewalks tagged as separate way or between the footway-road intersection nodes of a dual carriageway. Methods need to be developed in order to enrich the quality of OSM data with regard to such information. The motivation of this study is to prepare OSM data for the proper and efficient routing of people with restricted mobility (CAP4Access European project: http://www.geog.uniheidelberg.de/gis/cap4access_en.html) [11].

For this issue, in this paper, we specifically focus on the problem of how to support topological queries over features that are only implicitly defined. We present a geospatial rule-based reasoning approach for inferring geospatial objects in OSM. More specifically, we focus on Open Street Map as dataset and use access points to footways as a motivating example in the domain of routing and navigation. Geospatial information retrieval is an integral part of routing and navigation services, notably to help find the relevant landmarks and points of interests that should be displayed on the map or used as destination points [12,13,14]. Several approaches for retrieving landmarks or points of interest are able to process queries to retrieve entities that exist in the source, such as stadiums, hospitals, and lakes, etc. [15]. However, existing approaches (see Section 2) still have difficulties to resolve problems that require more details on geometries, topology and semantics. Notably, entities that are not explicitly stored as instances in the database cannot be retrieved. For example, consider an OSM user who wants to retrieve entry points of footways to plan for a hiking journey. While footways are explicit entities in OSM database, entry points of footways are not. The approach presented in our paper is based on the idea that spatial relations between explicit entities can reveal other implicit entities. Therefore, appropriate modeling can help to support reasoning with these relations and inferring the existence of implicit entities.

**Figure 1:**
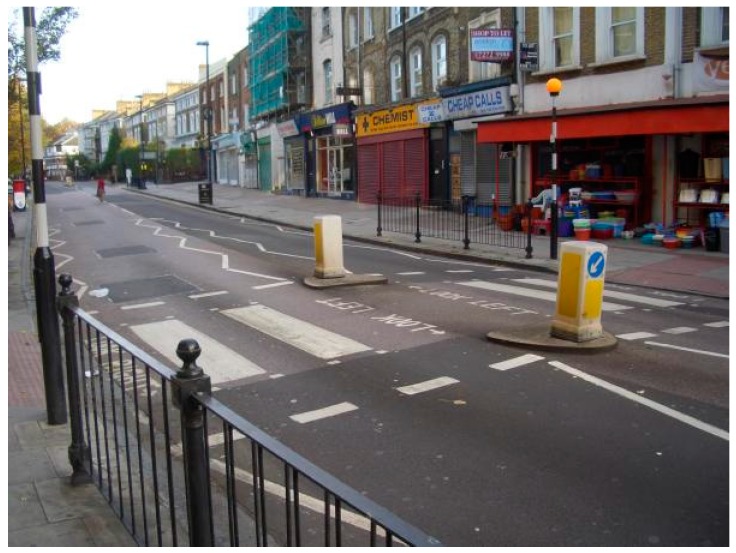
An example of footway crossing and its entry points. Photo credit: OpenStreetMap Wiki.

We have developed an information broker that uses the Semantic Query-Enhanced Web Rule Language (SQWRL). This language enables to identify entities that verify conditions specified with SWRL rules, which is the candidate rule language for the Semantic Web [16]. For example, in the case of our study, this language enables to state that “*if a footway intersects a street, then the intersection between the footway and the street is an entry point for the footway*” (Figure 1). However, this rule-based reasoning needs to be coupled with semantics of geo-spatial objects. In order to support the inference of such statements, we have implemented a spatial reasoning service based on an extended version of the Vertical Plane Sweeping algorithm to identify topological relations between spatial entities. In addition, we propose a framework where annotations and mappings between OSM data attributes and external resources, such as lightweight taxonomies, support the enrichment of queries to improve the ability of the system to retrieve information.

Furthermore, this article addresses the challenge of enriching quality of OSM data in terms of data completeness. We argue that the completeness of certain objects in OSM are low. There are missing objects that are required for wheelchair routing. The example is footway road crossings that are currently not mapped in the OSM database, but could be implicitly derived through spatial reasoning and analysis. Deriving this information leads to enriching the dataset with useful information, and thus enhancing the quality of OSM for wheelchair/pedestrian routing systems.

The structure of the paper is as follows. Section 2 presents related studies regarding OSM data quality enrichment, as well as selected methods of information retrieval and reasoning relevant to this study. In Section 3, the methodology and the system architecture of our proposed approach as well as spatial and semantic querying and reasoning algorithms are discussed. Section 4, shows the results of our experiments with outlining the experiences achieved. Finally, we conclude our study in Section 5 and discuss some ideas for future work on this topic.

## 2. Related Studies

Nowadays, users can produce geographic information via a variety of Internet applications. As a result, a “global digital commons of geographic knowledge” is created without having to rely solely on “traditional” geospatial data production processes [17]. In 2007, Goodchild introduced the term VGI to refer to the geographic information generated by users through Web 2.0 era applications [18]. VGI is often created out of the collaborative involvement of large communities of users in a common project—for example, Open Street Map (OSM) or Wikimapia (http://wikimapia.org)—where individuals can produce geographic information that emanates from their own local knowledge of a geographic reality or to edit information provided by other individuals. In OSM, users can describe map features—such as roads, water bodies, and points of interest—using “tags”, providing information with more attributes that often goes beyond the detailed dataset that can be provided by traditional geospatial data producers [19]. VGI datasets have been recently used in several studies in various applications domains, such as urban population estimation [20], cycling and air pollution exposure [21,22], three-dimensional (3D) GIS modeling of buildings [23], as well as routing and navigation services [24,25], to name a few. Hence, the availability of VGI data appears as an opportunity to improve various applications, including routing and navigation services. However, VGI data in itself is of no great value unless we find a means of managing and analyzing this less conventional data. As an example in our study, for the case of wheelchair routing and navigation, one would need to extract and use information, such as sidewalks or footway crossings, in order to make the most use of this dataset. However, such information are not explicitly mapped by the volunteers. Hence, the research question raised is how to extract information and knowledge from this raw and heterogeneous data?

Existing analytic techniques for extracting knowledge from data are being improved to be able to deal with massive datasets. These techniques include SQL queries, data mining, statistical analysis, clustering, natural language processing, text analytics, and artificial intelligence, to name a few [26]. Nevertheless, there is a general lack of semantics that would enable to process the existing data intelligently. Without semantics, one cannot reason on raw data to infer higher level facts, and therefore, to answer less obvious queries. Also, explicit semantics can help to filter data according to its meaning, which is really necessary if we cannot afford the cost of processing huge volumes of data. This lack of semantics notably affects VGI datasets [27,28,29]. The semantics of attributes of objects in OSM are important in this study, since it helps to perform geographical associations between certain objects, and thus, infer meaningful information.

Geospatial information retrieval aims at finding relevant geospatial information sets over distributed and heterogeneous data sources. Geospatial data retrieval approaches include, on the one hand, approaches that allow users to submit queries using their own vocabulary through a natural language interface. Such an approach has been proposed, for example, by Zhang et al. [30]. On the other hand, other geospatial data retrieval approaches enable the user to submit queries formulated only with primitives defined in an ontology, i.e., a formal specification of a conceptualization [31]. While natural language approaches allow users to submit more expressive queries than ontology-based approaches, natural language approaches are also restricted by the ambiguities of natural language, which may refrain from retrieving the relevant datasets [32]. In this paper, since our aim is not to focus on the resolution of ambiguities generated by natural language, we also adopt an ontology-based approach, such as those discussed below.

The Bremen University Semantic Translator for Enhanced Retrieval (BUSTER), proposed by [33], is an early example of ontology-based information broker middleware for geospatial data retrieval. This approach is representative of a category of retrieval approaches that have exploited Description Logics (DL) ontologies, such as [34] and [35] for means of collaborative development and usage of ontologies in GIScience domain [36]. Description Logics, which underlies the Ontology Web Language (OWL), allows for representing classes of individuals (entities) and properties. They also support subsumption reasoning, i.e., the automatic identification of sub-class relationships between classes. In the BUSTER approach, each data source’s semantics is formalized with a DL ontology. Each ontology is developed using a common vocabulary defined in a global ontology. The user can select the query concept from one of the ontologies or specify a query with necessary conditions (in term of properties and range of properties). The RACER and FaCT reasoning engines are used to retrieve the concepts that are subsumed by the query concept.

While the global ontology makes the different ontologies comparable to each other, assuming that local ontologies can be developed from a global ontology is not always feasible in an open and dynamic environment where sources are developed independently. Lutz and Klein [32] proposed a similar approach for the discovery and the retrieval of geographic information in Spatial Data Infrastructures. Their approach is also based on semantic annotations of geographic feature types with DL classes. The DL classes are compared with those that compose the user’s queries using a DL subsumption reasoning engine. Similarly to the BUSTER system, this approach retrieves only the classes that are subsumed by the classes in the query. This system does not allow for expressing complex queries with conditions as in the SQWRL language. Pursuing the work of [32,37] used the Semantic Web Rule Language (SWRL), a combination of OWL-DL with sublanguages of the Rule Markup Language (RuleML), to answer users’ queries over several data sources in SDIs. In this paper, we propose a geospatial data retrieval approach that builds on the foundations established in the latter approach, using the SQWRL query language. While [37] assumed that the semantics is shared by all requestors and providers (i.e., they use the same application ontology), in our approach, we do not make this assumption and we rather address the issue of employing ontologies by proposing a query enrichment approach based on a framework of semantic annotations and mappings among various resources. In addition to this first contribution, we propose an SWRL-based information retrieval approach that will enable the retrieval of implicit information, i.e., geospatial entities that do not exist in the available source, but which existence can be inferred from existing data. The usability of our approach for information retrieval is demonstrated in support of routing and navigation services. Our study aims to show the implication and possibility of using semantics and ontologies for the enrichment of OSM data completeness. Few studies have dealt with this topic and hence it could be mentioned that this study is one of the first attempts to address such a possibility. It is worth noting that another study [38] has also employed a rule-based reasoning approach to study OSM data quality. The authors have studied the dynamic patterns of OSM bugs in order to analyze and understand the reliability/quality of OSM database. In our study, however, we consider using rules and topological associations not for the assessment but, in order to derive new information and further enrich the quality of the dataset.

Furthermore, this study deals with learning from spatial relations between two or more objects. There have been several studies on this topic. Touya et al. [39] present an ontology of spatial relations and further show how spatial relations could be modeled for improving the consistency of datasets, as well as support automated processes. In another study [40], semantics of data coupled with spatial relation reasoning has been used to support and improve geo-positioning. For the OSM dataset, Corcoran et al. [41] propose a high level conceptual model of spatial relations. Similarly, they provide a use-case of spatial relation “enters” that may exist between a road and a housing estate, which is equivalent to highway = residential tag in OSM, and thus addressing the semantic/thematic accuracy of OSM. Our study differs in such a way that we propose an approach that generates new objects rather than deriving/inferring attributes of objects. Further details of our approach and its differences are provided in Section 3.

## 3. Methodology

**Figure 2:**
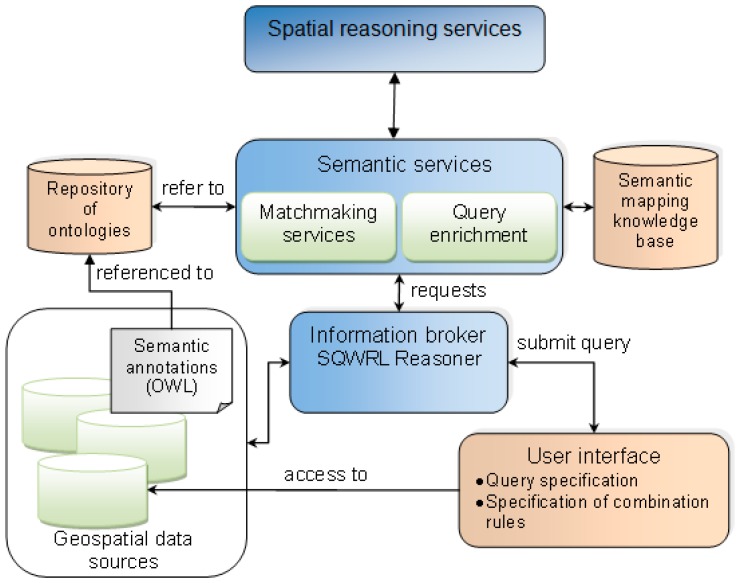
The conceptual architecture.

In order to deliver a geospatial reasoning approach that enables the retrieval of implicit information, i.e., geospatial entities that do not exist explicitly in the available source, we have developed an information broker that uses a rule-based spatial reasoning algorithm to detect topological relations. The information broker is embedded into a framework where annotations and mappings between OSM data attributes and external resources, such as taxonomies, support the enrichment of queries to improve the ability of the system to retrieve information. The system architecture is designed around the information broker, which is a mediator between the available geospatial data sources and the user who is seeking for information (Figure 2). Through the user interface, the user can specify a SQWRL query. The SQWRL query is processed with the Jess Rule Engine [42]. The matchmaking services produce the semantic mappings necessary to compare the query with the sources’ description. This system is based on principles of standard architectures for the retrieval of data or services, such as proposed by Vögele et al. [33] and Klien et al. [43]. However, the first contribution of the proposed approach with respect to existing work is to enhance the information broker with SQWRL to support the retrieval of implicit information. Through OWL and SQWRL rules, it is possible to specify relations between entities that will allow for the inferring of the existence of implicit entities. In order to retrieve implicit entities, we introduce a spatial reasoning service. The inference of implicit entities is based not only on semantics but also on spatial relations between existing entities stored in the data source. Therefore, the spatial reasoning service implements a spatial algorithm for identifying spatial overlap and adjacency of vector data, namely, the Vertical Plane Sweep technique. Please note that the architecture presented in Figure 2 is a conceptual architecture and not the exact architecture implemented in this study. However, an adopted simpler version of it is used for our experiments presented in Section 4.

In addition, in comparison to existing approaches, we do not assume that all of the sources are described according to the same application ontology or that the sources use a static terminology. Although this assumption facilitates retrieval, it is not realistic in the context where available sources describe different application domains. It is not also realistic in the context of VGI, where heterogeneous terminology is likely to be used. In order to address the issue of heterogeneous ontologies, as a second contribution, we introduce a query enrichment approach. In the following, we introduce the semantic annotations that support the query enrichment approach, presented in Section 3.2. The spatial reasoning is presented in Section 3.3.

### 3.1. Semantic Annotations

Semantic annotations are defined by Klien [44] as explicit correspondences (mappings) between the components (classes, attributes, relations, values, etc.) of the data schema of a source and the components (classes, properties, etc.) of an ontology. We also consider that semantic annotations include correspondences between components of an application-specific ontology and components of a more general reference ontology. Semantic annotations enable reasoning with the semantics without altering the local data schemas of sources or application ontology. In this approach, we choose to store semantic annotations in a separate source, since the method allows for using a controlled ontology (either domain or reference). A semantic annotation is formed by a pair of unique identifiers of components from a local source and an application ontology. This association means that the ontology component is the formal representation of the semantics of the local sources component. Because semantic annotations are used to infer which sources contains elements that match a SQWRL query, semantics annotations are formalized with OWL.

### 3.2. Semantic Querying

**Figure 3:**
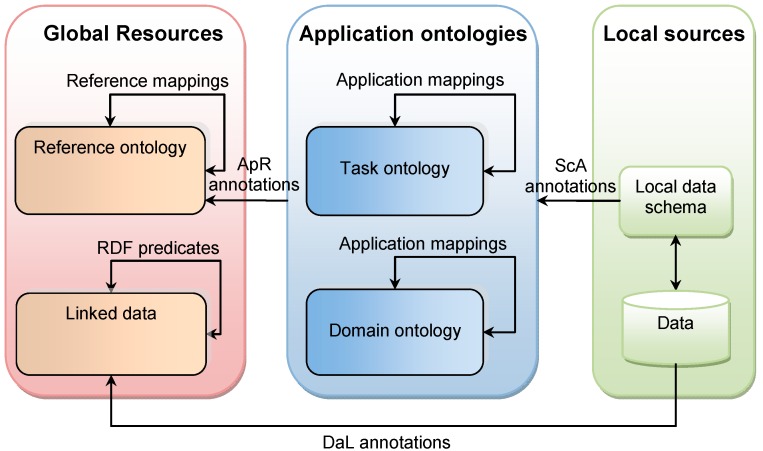
System of resources, semantic annotations and mappings supporting query enrichment. ScA: schema-to-application ontology annotations, ApR: application-to-reference annotations.

The principle of query enrichment is to expand the elements of the query (which are ontology components or values) with other elements that use a different terminology but have the same meaning. This approach is based on methods for information retrieval described by Boghal et al. [45], as techniques using “corpus-independent knowledge models”, in comparison with approaches that apply knowledge extraction techniques to a set of documents to enrich a query. In the ideal case, the equivalence of meaning is established through a system of semantic annotations and semantic mappings among various resources (Figure 3). Please note that Figure 3 shows the conceptual mapping of concepts between various sources and is not necessarily implemented in this study. However, the general concept is valid and is adopted in our study.

The resources are situated at three levels, i.e., local sources, applications ontologies, and global resources. Application ontologies include domain ontologies (describing a knowledge domain, such as ecology, health, etc.) and task ontologies (designed to support the execution of some activity, such as land use management, disaster planning, etc.). Global resources include reference ontologies, which are domain- and application-independent ontologies, and Linked Data. Linked Data is a Web of data coming from different sources, linked through Re-source Description Framework (RDF) predicates [46].

Semantic mappings link components from the same level, while semantic annotations link components from different levels. Components of local sources’ data schemas are linked to components of applications ontologies through schema-to-application ontology annotations (ScA annotations, stored in the ScA Annotation Knowledge Base (KB)) (Figure 3). Components of application ontologies are linked to components of reference ontologies through application-to-reference annotations (ApR annotations, stored in the ApR Annotation Knowledge Base). Data from local sources can be linked to URIs on Linked Data through so-called DaL annotations (stored in the DaL Annotation Knowledge Base) (Figure 3).

Semantic mappings between ontologies, ScA and ApR annotations support the enrichment of the ontology components that compose queries (classes and properties), while DaL annotations support the enrichment of the values that compose queries. The query enrichment algorithm (Algorithm 1), uses mappings and annotations to retrieve elements that can be substituted to components of the query. In this way, a query can be substituted by a set of equivalent queries that use equivalent terms of different ontologies. The enrichment can be horizontal, i.e., a component of a query (which is a component of an application ontology) is replaced with a component of another application ontology, if a semantic mapping that links these components exists. The enrichment is vertical when a component of a query is replaced with a component of a reference ontology, as identified through an ApR annotation. The semantic mappings, which are stored in knowledge bases, can be established manually or through a semantic matchmaking service. For example, Bakillah and Mostafavi [47] have provided a semantic mapping system that can help to support this matching task.

**Algorithm 1.** Query Enrichment Algorithm**Enrich (query *q*): List <query>**1:Declare and initialize a list of queries
*equivalent_Query*2:Add *q* to *equivalent_Query*3:For all elements *el* of *q*4:  **If *el* is an ontology component**5:    Access Application Mapping KB6:      For all mappings *m* where *el* is a participant7:       Get the relation *r* stated by *m*8:       If *r* == equal9:           Create a copy *q*’ of *q*10:         Get *el*’, the appl. onto. component linked to *el* through *r*11:             Replace *el* with *el*’ and direct sub-concepts of *el*’ in *q*’12:           Add *q*’ to *equivalent_Query*13:    Access ApR Annotation KB14:      For all ApR annotations *a* where *el* is a participant15:        Get *el*’, the reference onto. component linked to *el* through *a*16:         For all ApR annotations *a*’ where *el*’ is a participant17:             Get all appl. onto. components *c* linked to *el*’ through *a*’18:            For all appl. onto. components *c* linked to *el*’ through *a*’19:              Create a copy *q*’ of *q*20:              Replace *el* with *c* and direct sub-concept of *c* in *q*’21:              Add *q*’ to *equivalent_Query*22:  **If *el* is a value**23:    Access DaL Annotation KB24:    For all DaL annotations *a* where *el* is a participant25:       Get *el*’, the name of the Linked Data component linked to *el* through *a*26:       Create a copy *q*’ of *q*27:       Replace *el* with *el*’ in *q*’28:       Add *q*’ to *equivalent_Query*29:  Return *equivalent_Query*

### 3.3. Spatial Reasoning

Implicit geospatial entities can be identified from spatial relations between two other explicit entities. For example, if we have two polygons representing two States, we can infer that the intersection line is the “border”. In order to reveal the existence of such implicit entities, two conditions must be fulfilled: (1) the implicit entity is semantically modeled according to the relation between two (or more) other entities; and, (2) a spatial reasoning algorithm can compute spatial relations between entities. Condition 1 can be fulfilled by modeling the relations with OWL and SQWRL. An example is provided in the case study of Section 5. As for condition 2, we employ the Vertical Plane Sweeping technique presented in [48].

The Vertical Plane Sweeping technique applies to vector data where polygons are represented by their edges. Therefore, it is suitable for the OSM dataset where entities are formed by edges and points. The Vertical Plane Sweeping technique enables to find the polygon that represents the overlapping regions between two polygons. Basically, in order to find this overlapping region, the algorithm first find the intersection points of the two polygons using the intersection algorithm described in [49]. Then, in order to subdivide the edges of the polygons at intersecting points, it is supposed that the plane is swept with a vertical line. Every time the sweep line reaches an edge, this edge is added at the top of a dynamic list of edges. It was demonstrated that the two following statements are true: (1) the list is amended only when the sweep line reaches the endpoint of an edge or the intersection of two edges; and, (2) only edges that are adjacent in the list can intersect in space. From then, the edges that form the overlapping region can be identified. In this paper, finding the overlapping region is useful to identify spatial relation between two entities but we also need to be able to identify adjacency or “quasi-adjacency”. Indeed, due to a possible lack of positional accuracy, it is possible that two entities that have no common coordinates in the database can still overlap in the reality. For example, if a footway’s endpoint is almost adjacent to a road in the database (lets say at one meter distance), it is likely that in the reality the footway can be accessed from the road, and in fact, they intersect. Therefore, we extend the algorithm to include this case of “quasi-adjacency”.

The following algorithm (Algorithm 2) is the algorithm presented in [48] extended with procedure to detect quasi-adjacency. Q is the list of endpoints that form the edges of both polygons. Event is used to represent intersection of sweep line with the endpoint of an edge (and correspond to coordinates). The variable S represents the dynamic list of edges generated as the vertical line sweeps the plane. If the result of the Vertical Plane Sweeping algorithm is no overlap (list of intersection edges is empty), the minimal distance between the two polygons is computed. If the minimal distance is less than a selected threshold, we consider the polygons to be quasi-adjacent. In these conditions, we also consider that there is an intersection point between the two entities.

**Algorithm 2.** Extended Vertical Plane Sweeping1:  Insert the endpoints of the edges of polygons into list of endpoints Q2:  while (! Q.empty ()) {3:    event = Q.top ();4:    Q.pop ();5:      if (event.left_endpoint ()) { 6:      pos = S.insert (event); 7:      event.setInsideOtherPolygonFlag (S.prev (pos)); 8:      possibleInter (pos, S.next (pos)); 9:      possibleInter (pos, S.prev (pos));10:      } else { // the event is a right endpoint11:    pos = S.find (*event.other); 12:    next = S.next (pos); 13:    prev = S.prev (pos); 14:      if (event.insideOtherPolygon ()) Intersection.add (event.segment ());15:      if (! event.insideOtherPolygon ()) Union.add (event.segment ());16:    S.erase (pos); 17:    possibleInter (prev, next);18:          }19:    }20:  If Intersection.empty()==true { //the polygons are not overlapping21:    minimalDistance = GetMinimalDistance(Q);22:      If minimalDistance <= DistanceThreshold {23:      quasiAdjacent(Q) = true;24:      }25:  }

## 4. Experiment, Results and Discussion

**Figure 4:**
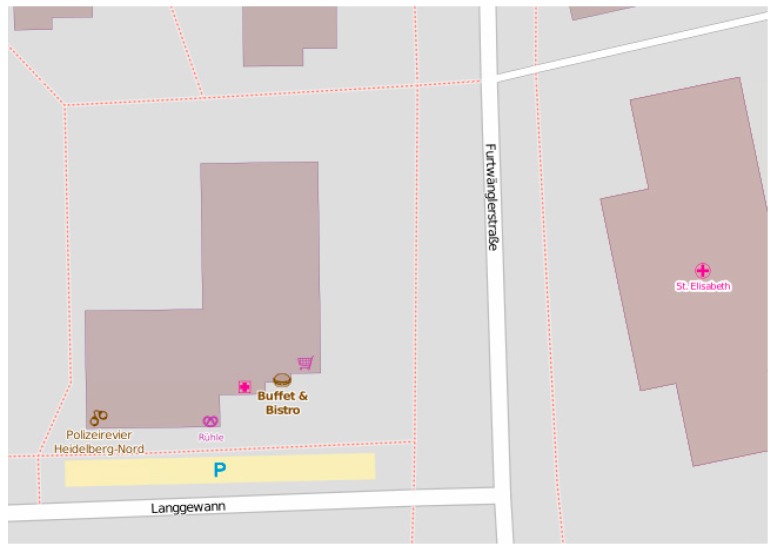
Footways are displayed as dotted lines but entry points of footways are not easy to identify.

In order to show the possibility of employing ontologies for OSM data reasoning and enrichment, we have implemented and tested our proposed methodology with two case studies. It is important to note that this study does not deal with addressing the issue of heterogeneous ontologies, and the architectures provided in Figure 2 and Figure 3 are proposed as a general solution for this issue. As a first case study, consider a user with impaired mobility who wants to plan some travel using OSM. The user wants to easily find the entry points of footway in a given area. Unfortunately, footways are represented as segments, but footway entry points are not explicitly identified in OSM. This is illustrated on Figure 4, where footways are represented with dotted lines. It is difficult to visually tell where the entry points of footways are. There could be entry points at the intersection of the footway and Langgewan Str., at the intersection of the footway and Furtwängler Str., etc. However, the user cannot be sure, since there could be a bridge, or any type of barrier at the apparent intersection point that could make the footway inaccessible at that point. Most routing and navigation maps would not be able to identify entry points of footways (among other similar difficulties) and we cannot assume that they are easy to detect by just looking at the map. We demonstrate how our proposed methodology can help to resolve this problem.

**Figure 5:**
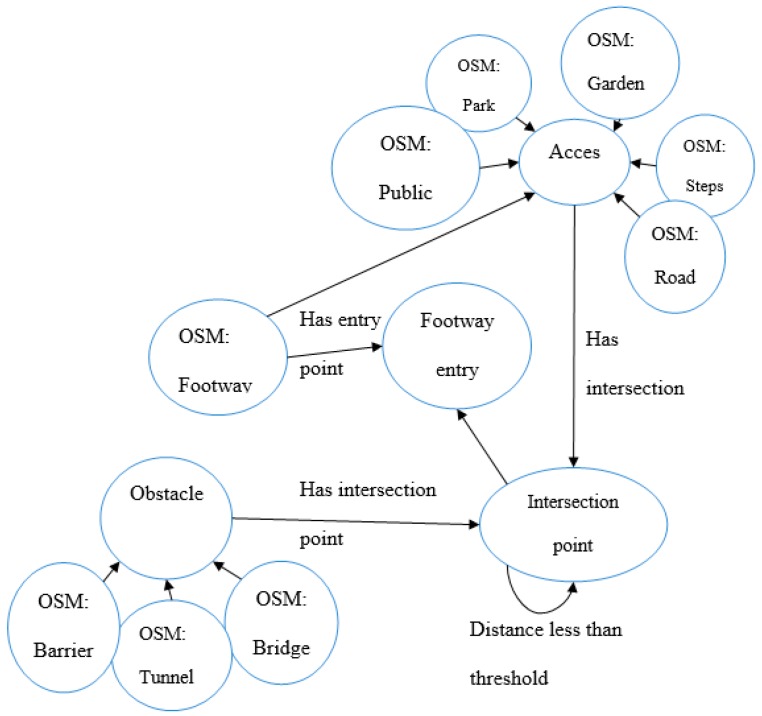
Ontology Web Language (OWL) ontology model to support the retrieval of footway entry points.

To start with, the entity “footway entry point” and its relations with other types of entities in OSM have to be modeled to support the reasoning process. For this scenario, we have developed the OWL ontology model, as illustrated on Figure 5. The model contains two types of entities: entities that exist in OSM (identified with prefix: OSM), as provided by the recommended terminology for tags (http://wiki.openstreetmap.org/wiki/Map_Features), and entities that were added to support the reasoning process. These added entities are:

- “footway entry point”, the feature the user is looking for;
- “intersection point”, which represent the coordinates of the intersection between two entities, such as a footway and a road;
- “Access area”, which represents any OSM entity from which footways can be accessed, for example, a park, a garden, steps, etc. For the sake of simplicity of the figure, only some entities are represented here, but more entities were taken into account;
- “Obstacle”, which represents any OSM entity that can be an obstacle to accessing a footway. Similarly, only some entities are represented here, but more entities were taken into account.

In addition to entities, relations were created to support the reasoning. Access areas and obstacles can have intersection points with each other. The identification of these intersection points is the first step towards finding entry points of footways. In addition, the distance between intersection points is explicitly modeled and will be useful, as explained below, to discard intersections points that will not be considered as footway entry points. First, we assume that the user can select a buffer zone (denoted as Z1, representing a shapefile) that represents the area of interest. This buffer can be set to any value depending on the case study, and is applied to layers that provide the data that needs to be queried (e.g., road network). The user’s query for retrieving footway entry points is formulated as a SQWRL query:

**Query:**  FootwayEntryPoint(?P)∧SelectedBufferZone(Z1)∧Inside(?P,Z1)

→sqwrl:select(?P).

We assume that, while “footway” is OSM’s recommended term, because VGI is intrinsically heterogeneous, other similar terms could have been used to refer to the same category of entities. Therefore, the query statement FootwayEntryPoint(?P) is enriched as follows with WordNet entries retrieved from semantic annotations (Φ implication symbol is employed for enrichment):

**Enrichment:**  FootwayEntryPoint(?P) Φ (FootwayEntryPoint Φ Path Φ Way Φ Hiking)(?P).

Using the relation “Has entry point”, the following query is generated and processed to retrieve all of the footways that overlap or are adjacent with the buffer zone Z1 with the extended vertical sweeping algorithm:

**Query:**  Footway (?F)∧SelectedBufferZone(Z1)∧Overlap(?F,Z1) →sqwrl:select(?F).

Then, for each retrieved footway, we need to find their intersection(s) with access areas to find potential entry points. However, it would be costly in terms of processing to check the intersection between footways and all entities considered as access areas in the buffer zone Z1. Therefore, for each retrieved footway in Z1, we generate the minimal buffer zone that includes the segments forming the footway. Let Z2 be a minimal buffer zone. This operation results in the generation of a series of statements of the following form, which are stored as semantic annotations:

**Statement:**  FeatureBufferZone(Z2).

The next step is to retrieve all instances of access areas that overlap with the minimal buffer zone Z2 (for each minimal buffer zone computed):

**Query:**  AccessArea(?a) ∧ FeatureBufferZone(Z2) ∧ Overlap(?a, Z2) →sqwrl:select(?a).

In fact, this query is rewritten with the help of the “is-a” relation in the ontology model to be able to process it against OSM data:

**Query:**  [publicTransport Φ park Φ garden Φ steps Φ road Φ …](?a) ∧ FeatureBufferZone(Z2) ∧ Overlap(?a, Z2) →sqwrl:select(?a).

Furthermore, the elements of the query are semantically enriched to include similar terms. Then, the extended vertical sweeping algorithm is used to identify the intersection points between a footway and the access areas that were detected within its minimal buffer zone. As a result, a set of statements of the following form are generated as semantic annotations:

**Statement:**  IntersectionPoint(I)

**Statement:**  HasIntersectionPoint(F, I)

These intersection points between a footway and an access area are only potential points of entry to footways. In some cases, some could not be entry points because at the same place, or very close, there is an obstacle that refrains from accessing the footway. Therefore, the following query is generated:

**Query:**  IntersectionPoint(I?) ∧ Footway(?F) ∧ HasIntersectionPoint(?F, ?I) ∧ Φ [IntersectionPoint(Q?) ∧ Footway(?F) ∧ HasIntersectionPoint(?F, ?Q) ∧ Obstacle(?o) ∧ HasIntersectionPoint(?o, ?Q) ∧ DistanceLessThanThreshold(?Q, ?I)] →FootwayEntryPoint(?I)

**Figure 6:**
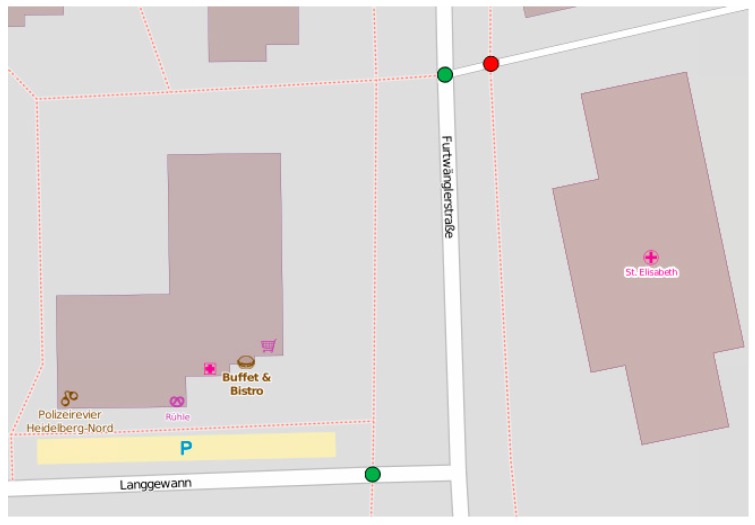
Query results identifying the entry points of footways. Identified entry points (in green) and discarded intersection points (in red).

It adds a clause that says that an intersection point I of footway F is considered as an entry point of this footway only if there exists no other intersection point Q between the footway and an entity of the category “obstacle” that lies within a distance of less than a given threshold from I. In the case of the above query, we have considered a threshold of 5 m to take into account the lack of accuracy of positioning of features in OSM. This allows for discarding intersection points where there is no access in reality. In Figure 6, as a result of this query, we can see the entry points that were identified (in green) and the intersection points that were discarded (in red) following this principle.

**Figure 7:**
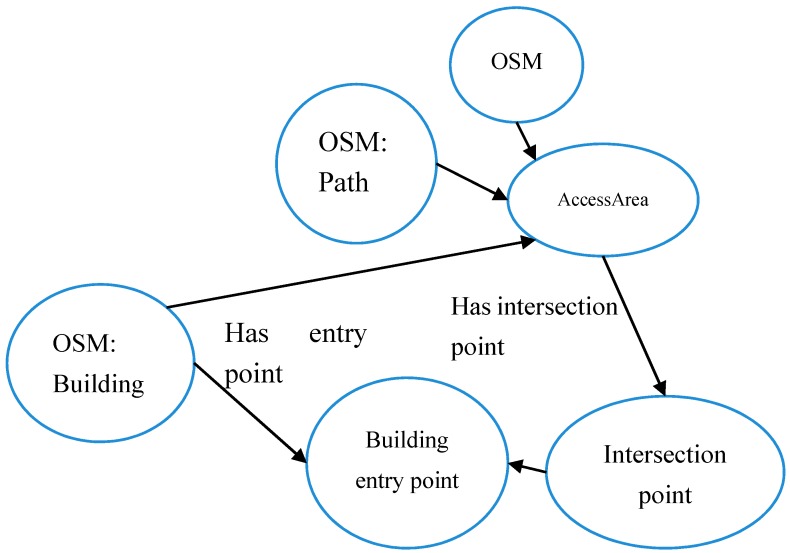
OWL ontology model of spatial relations between buildings and access areas (paths or steps).

As another similar example where spatial relations enable the retrieval of implicit spatial entities in OSM we employ our approach to derive entry points of buildings in OSM. Building entry points are defined as the intersection point between paths and building footprints. Although this assumption might not always be true, but the authors believe that in most of the cases this could be the real situation. They are necessary in order to provide the possibility of suggesting efficient navigation guides by the routing services, especially in the case of integrating outdoor and indoor navigation. In this regard, it is clear that entry points of buildings are missing in OSM database. In order to identify implicit entry points, a spatial relation between buildings and “paths” was exploited, i.e., when building and path intersect it was inferred that entries exist at this intersection point. The OWL ontology model for this relation is identified in Figure 7. In this case, the access area can be paths or steps. Figure 11 shows an example of the results of the query for building entry points.

**Figure 8:**
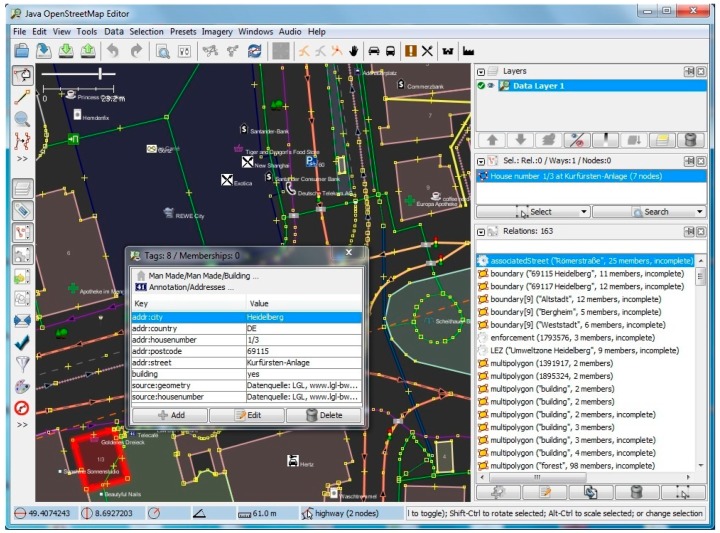
Relational properties of a sample random building.

**Figure 9:**
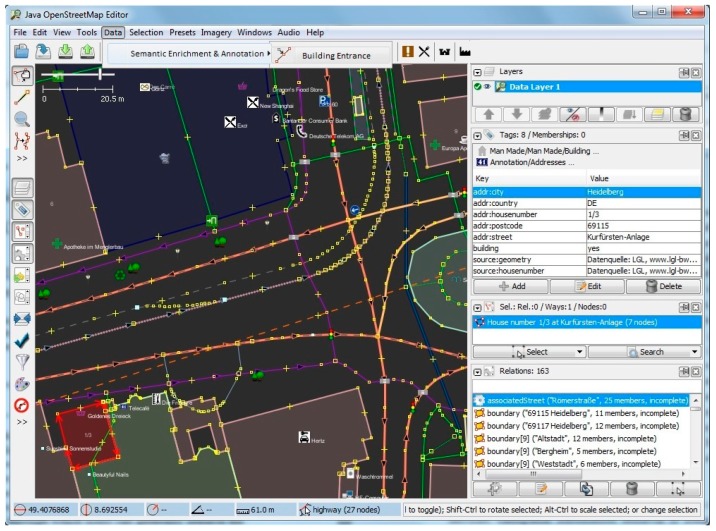
Semantic Enrichment & Annotation plug-in menu in Java OpenStreetMap (JOSM).

**Figure 10:**
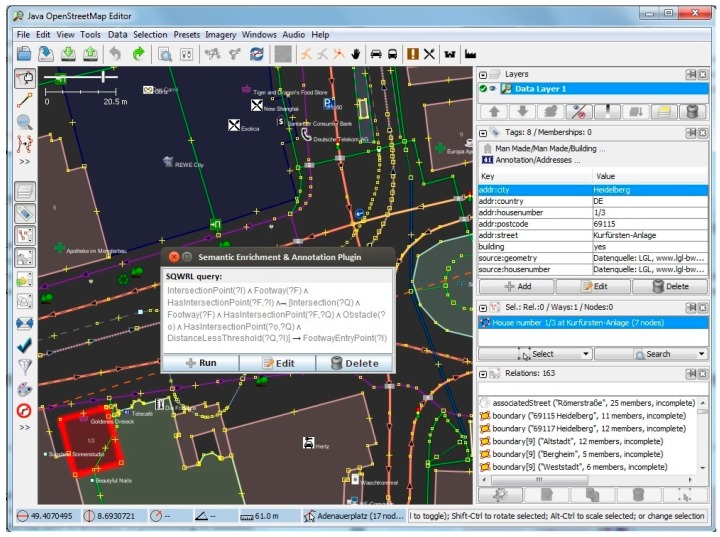
Enriched Semantic Query-Enhanced Web Rule Language (SQWRL) query to derive building entrances.

**Figure 11:**
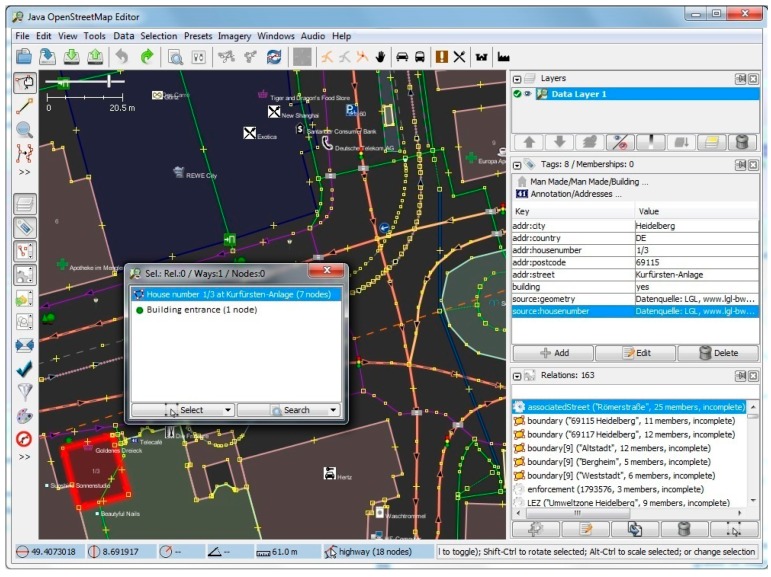
Resulting building entry node (green circle) in the relationship properties menu of the selected building.

Furthermore, we have implemented and tested our method for a district in Heidelberg (area containing 228 road segments and 87 buildings), and have evaluated the results of the footway intersections and building entrances with visual checking in fieldwork. Figure 8, Figure 9, Figure 10 and Figure 11 show screenshots of the experiment and development stage in Java OpenStreetMap (JOSM) Editor. JOSM is the most commonly used OSM editor. It is a free, open source and stand-alone desktop application that allows contributors to create, edit, or delete data from OSM. Figure 8 depicts the properties page for a selected random building with 8 tags and 0 memberships. On the right-hand side panel, details of the relations such as the boundaries as well as the associated street are also extracted and shown. Figure 9 shows the usage of the plugin for semantic enrichment and annotation. Moreover, Figure 10 shows the dialog box for deriving (and if needed, editing) the Enriched SQWRL query to derive building entrances, and finally in Figure 11 the result of executing the query and deriving the building entry nodes is shown.

For evaluating the results, we have performed the experiments for a district in Heidelberg and have visited the field for ground-truthing the results. For the footway intersection points, in terms of completeness, there were only 9 out of 217 excessive nodes, which were caused by some topological inconsistencies of OSM data for the area. Moreover, we found eight footway intersection points that were not discovered by our algorithm. The main reason for this was the incompleteness of footway data in OSM for that specific area. Since our approach analyzes the footprints as well as road data in OSM, missing this data for an area would logically lead to lack of functionality in our approach. In terms of positional accuracy, it reaches an approximate average accuracy of half a meter (0.47 m) as compared to ground truth, in which part of this inaccuracy could have also been propagated through the errors of the original OSM dataset itself. This level of accuracy is acceptable given the fact that the dataset would later be used by a routing and navigation service that provides instructions prior to traveling, and not necessarily at the exact time of travel. In the latter case, however, one can still argue that the level of accuracy of the results are acceptable. For the second case scenario, in a total amount of 87 buildings (with 92 entrances), the algorithm was not able to predict entrances of 5 buildings where the building footprints were lacking. The average positional accuracy of entrances for the other 82 buildings were less than 1 m.

Our experiment illustrates how useful it can be to employ spatial relations to infer the existence of implicit geospatial entities during information retrieval. It also shows that while semantics of crowdsourced data such as in OSM can be poor, semantic approaches can be employed to infer more information from data already available. This is especially useful assuming that it would not be realistic to expect OSM contributors to provide more detailed semantics. More detailed and explicit entities would also have as a result to increase the volume of data, which would be more costly to process. In contrast, in the proposed approach, a routing and navigation application could avoid being overloaded by huge volumes of data, but when additional information not available in the database is required (e.g., footway intersections), it can be inferred from existing data on a case-by-case basis (based on the user’s interest), provided that an ontological model of the implicit entities (as in Figure 5 and Figure 7) exists. It is important to note that employing semantics and ontologies for this task provides the possibility of further improving this system by making it smarter, in terms of using heterogeneous ontologies, integrating it with other data sources that could help in the enrichment of the data quality, etc. While this issue is not addressed in this study, nevertheless the idea of using ontologies provides such functionality as compared to simple analysis on single data sources (relational, object-relational databases).

## 5. Conclusions and Future Work

This study aimed to show the relevancy and applicability of using semantic technology and spatial reasoning for OSM data enrichment. We have addressed the issue of retrieving implicit geospatial information from VGI sources, namely Open Street Map. We argued that research should be conducted to improve the ability of geospatial information retrieval techniques to retrieve implicit information that can be extracted from existing data. Following this idea, we proposed a geospatial information retrieval approach that uses the OWL and SQWRL language to model implicit entities based on spatial relations between existing entities. We have included this approach into an information broker that uses a set of semantic annotations to reason with semantics of data, whether explicit or implicit. The case study presented and the results with a scenario useful for routing and navigation service, in particular, shows the potential of this approach to answer different types of queries for information retrieval. Therefore, more semantics is not contradicting with the paradigm of Big Data, because it allows to keep datasets less voluminous by avoiding the generation of all entities as explicit instances in the database. Finally, it is concluded that this approach heavily relies on data availability (building footprints, road network data). The approach cannot be used in areas that miss the required data. However, this is a logical due to the fact that our approach is an intrinsic approach that relies on the existing data itself, and other sources of geo-data are not used in our method.

Nevertheless, in future work, we still aim to further investigate how Big Data technologies can help to make this approach applicable to massive datasets. For example, the ability to deal with massive datasets is supported by underlying technologies, such as Google’s MapReduce Big Data processing framework and its open-source implementation, Hadoop, which is now considered by some as a de facto standard in industry and academia. With MapReduce, data mining algorithms such as clustering, frequent pattern mining, classifiers, and graph analysis can be parallelized to be able to deal with massive datasets. In future work, we aim to explore how such technologies can improve our approach in terms of processing cost. In addition, we also plan to demonstrate that data from different sources can be merged to process queries on implicit entities. Among other examples, the picture portal Flickr can be used [50,51] to identify entities that are not explicit in the main dataset. Last but not the least, further studies regarding extending the modelling of spatial relations and the spatial reasoning services seem to be crucial. As another point for future research, it is believed that implementing semantic add-ins in JOSM that connects to OSM ontology [27] or other ontology resources would help highly in improving/controlling the quality of OpenStreetMap. This could be done in such a way to improve the existing tagging services [52,53] with ontologies and recommender systems. Therefore, we aim to apply our method on a bigger study area (city or country level) later when the method is concrete.
